# Ca_v_1.4 L-Type Calcium Channels Contribute to Calpain Activation in Degenerating Photoreceptors of *rd1* Mice

**DOI:** 10.1371/journal.pone.0156974

**Published:** 2016-06-07

**Authors:** Christian Schön, François Paquet-Durand, Stylianos Michalakis

**Affiliations:** 1 Center for Integrated Protein Science Munich CiPSM at the Department of Pharmacy – Center for Drug Research, Ludwig-Maximilians-Universität München, Munich, Germany; 2 Division of Ocular Neurodegeneration, Institute for Ophthalmic Research, Centre for Ophthalmology, University of Tübingen, Schleichstr. 4/3, 72076, Tübingen, Germany; Dalhousie University, CANADA

## Abstract

Retinitis pigmentosa is an inherited blinding disorder characterized by progressive degeneration and loss of photoreceptors. The exact mechanism of degeneration and cell death of photoreceptors is not known, but is thought to involve disturbed Ca^2+^—signaling. Ca^2+^ can enter the photoreceptor cell via outer segment cyclic nucleotide-gated (CNG) channels or synaptic Ca_v_1.4 L-type voltage-gated calcium channels (VGCC). Previously, we have shown that genetic ablation of the *Cngb1* gene encoding the B subunit of the rod CNG channel delays the fast progressing degeneration in the *rd1* mutant mouse model of retinitis pigmentosa. In this study, we crossbred *rd1* mice with the *Cacna1f*-deficient mouse lacking the Ca_v_1.4 α1 subunit of the L-type VGCC. Longitudinal *in vivo* examinations of photoreceptor layer thickness by optical coherence tomography revealed a significant, but not sustained delay of retinal degeneration in *Cacna1f* x *rd1* double mutant mice compared to *rd1* mice. This was accompanied by a reduction of TUNEL positive cells in the early phase of rod degeneration. Remarkably, *Cacna1f* x *rd1* double mutant mice displayed a strong decrease in the activation of the Ca^2+^-dependent protease calpain during photoreceptor loss. Our results show that genetic deletion of the synaptic Ca_v_1.4 L-type VGCCs impairs calpain activation and leads to a short-term preservation of photoreceptors in the *rd1* mouse.

## Introduction

Retinitis pigmentosa (RP) is characterized by a primary loss of rod photoreceptor function and structure. Subsequent non-cell autonomous degenerative processes eventually affect cone function and structure resulting in compromised daylight vision and in severe cases legal blindness.

The most extensively studied RP model is the *rd1* mutant mouse [1]—a naturally occurring strain expressing a loss-of-function mutation in the *Pde6b* gene encoding the β-subunit of the rod-specific cGMP phosphodiesterase 6 [2]. This mutation results in a fast progressive degeneration of rod photoreceptors during postnatal development of the retina. During pathogenesis, several important processes, including non-apoptotic, cell-intrinsic factors, trigger photoreceptor death [3]. One known key player is the elevated level of intracellular Ca^2+^ in degenerating photoreceptors of *rd1* mutant mice [4]. As a consequence, increased activity levels of the Ca^2+^—dependent protease calpain were found in different models of photoreceptor degeneration [3]. Ca^2+^ and calpain are therefore considered as potential executers of photoreceptor cell death. Currently, two different types of ion channels are known to mediate Ca^2+^—influx into photoreceptors: the cyclic nucleotide-gated (CNG) channel found in the outer segment plasma membrane [5] and the synaptic L-type voltage-gated Ca^2+^ channel (VGCC) [6, 7]. Given the importance of Ca^2+^—influx for photoreceptor degeneration [8], the Ca^2+^-conducting CNG channels and VGCCs were proposed as potential therapeutic targets for RP [4, 9]. However, pharmacological attempts to block Ca^2+^ influx have led to contradicting findings [8–13]. Genetic studies helped clarifying this discrepancy and showed that the knockout of the *Cngb1* gene encoding for the β1-subunit of the rod CNG channel significantly delayed photoreceptor degeneration in *rd1*^*mt/mt*^ mice [14, 15]. Genetic deletion of *Cacnb2* encoding the β2-subunit of the VGCC also resulted in a significant, but only transient decrease in the rate of photoreceptor degeneration in *rd1*^*mt/mt*^ mice (15).

In photoreceptors, the β2-subunit was suggested as the preferred β-subunit associated with Ca_v_1.4 channels [16, 17]. However, the pore-forming Ca_v_1.4 α1 subunit can in principle also functionally associate with other β-subunits [18, 19] which might also exist in the retina.

To define the role of the pore-forming Ca_v_1.4 α1 subunit of the VGCC channel on photoreceptor degeneration, we cross-bred the *rd1* model with *Cacna1f*-deficient mice [20]. We found that genetic deletion of *Cacna1f* led to a significant, but only short-term delay of photoreceptor degeneration in *rd1* mice. However, knockout of *Cacna1f* prevented excess activation of calpain commonly seen in degenerating photoreceptors (3), highlighting the dependence of this phenomenon on synaptic VGCCs.

## Material and Methods

### Ethics statement

The mouse studies were approved by the local authority (Regierung von Oberbayern, Az. 55.2-1-54-2532-50-15) and were conducted in accordance with the ARVO Statement for the Use of Animals in Ophthalmic and Vision Research. Mice were anesthetized by intraperitoneal injections of ketamine (0.05 mg/g) and xylazine (0.01 mg/g) and were sacrificed by cervical dislocation. All efforts were taken to minimize suffering of the animals.

### Animals

In this study, we compared *Pde6b*^*rd1*^ mutant mice (*rd1*^mt/mt^) with double mutant mice carrying the homozygous *rd1* mutation and lacking expression of the X-chromosomal *Cacna1f* gene (*rd1*^mt/mt^ x *Cacna1f*^ko/ko^). *Cacna1f*^ko/ko^ mice were provided by Dr Marion Maw, University of Otago, Dunedin, New Zealand [20]. Due to the lack of the pore-forming Ca_v_1.4 α1 subunit of the VGCC these mice develop a rod and cone photoreceptor synaptopathy and cone but not rod photoreceptor degeneration [21, 22].

### Optical coherence tomography (OCT) measurements

Before the scanning procedure, mice were anesthetized and Tropicamide eye drops were applied to the animals’ eyes for pupil dilation (Mydriadicum Stulln, Pharma Stulln GmbH, Stulln, Germany). Subsequently, hydroxylpropyl methylcellulose (Methocel 2%; OmniVision, Puchheim, Germany) was applied to keep the eyes moist. The OCT examinations were performed with a MICRON IV system (Phoenix research labs, Pleasanton, United States). A vertical OCT scan was centered to the optic nerve head and measurements were conducted in the dorsal part of the retina. InSight software (Phoenix research labs) was used to measure the photoreceptor layer thickness as the distance between the outer plexiform layer and the border of neuronal retina and the retinal pigment epithelium. The photoreceptor layer thickness as defined in our study is also known as “photoreceptor plus” or REC + [23].

### Calpain activity assay

To test for calpain activity an enzymatic *in situ* assay was used [24]. Unfixed retinal cryosections were pre-incubated for 15 min in calpain reaction buffer (CRB; 25 mM HEPES, 65 mM KCl, 2 mM MgCl2, 1,5 mM CaCl2, 2 mM DTT) and then incubated at 35°C for 1 hour in the dark in CRB with 2 mM fluorescent calpain substrate 7-amino-4-chloromethylcoumarin, t-BOC-Leucyl-L-methionine amide (CMAC, t-BOC-Leu-Met; Molecular Probes, Eugene, USA). Calpain-dependent cleavage of the substrate led to the uncaging of fluorescence, which was then used for quantifying the numbers of calpain activity positive cells.

### TUNEL assay

The terminal deoxynucleotidyl transferase dUTP nick end labelling (TUNEL) assay was performed using an *in situ* cell death detection kit (Fluorescein or TMR; Roche Diagnostics GmbH, Mannheim, Germany). As negative control the terminal deoxynucleotidyl transferase enzyme was omitted from the labelling solution, for positive control the sections were pre-treated for 30 min with DNAse I (Roche, 3 U/ml) in 50 mM Tris-HCl, pH 7.5, 1 mg/ml BSA to induce DNA strand breaks. Negative control gave no staining, while positive control stained all nuclei in all layers of the retina [25].

### Microscopy and cell counting

Fluorescence microscopy was performed on a Z1 ApoTome Microscope equipped with a Zeiss Axiocam digital camera (Zeiss, Oberkochen, Germany). Images were captured using Zeiss Axiovision 4.7 software and representative pictures were taken from central areas of the retina. Adobe Photoshop CS3 (Adobe Systems Incorporated, San Jose, CA) was used for image processing and figure assemblies.

For cell quantification, pictures were captured on whole radial sections using the Mosaix mode in Axiovision 4.7. Labelled cells (TUNEL, calpain activity) were counted manually. The total number of cells was determined by dividing the area of the outer nuclear layer (ONL) through the average ONL cell size. The number of positive cells was then divided by the total number of ONL cells to give the percentage of positive cells.

### Statistical analysis

Statistical analysis was performed using Graph Pad Prism 5 software. To compare groups at one time-point, either Welsh's t-test or one-way ANOVA with Tukey post-test was applied. For comparing groups in longitudinal OCT examinations, two-way ANOVA with Bonferroni post-tests were performed. All values are given as mean ± SEM and n indicates the number of eyes.

## Results

### *Cacna1f* knockout delays photoreceptor cell death in *rd1*^mt/mt^ mice after eye opening

**Fig 1:**
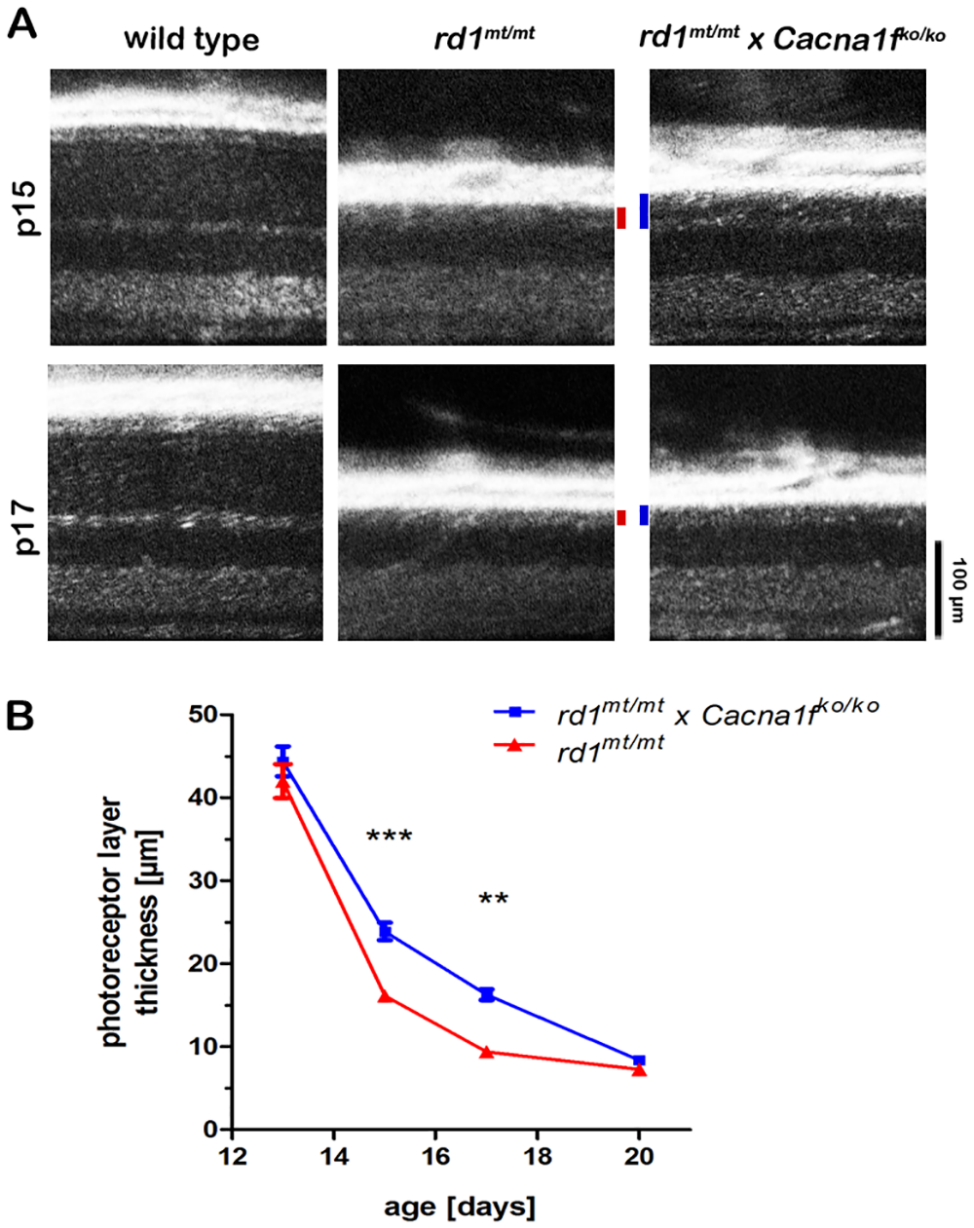
Knockout of *Cacna1f* delays photoreceptor cell loss in *rd1*^*mt/mt*^ mutant mice. (A) Representative *in vivo* OCT images of wild type, *rd1*^*mt/mt*^ *and rd1*^*mt/mt*^ x *Cacna1f*^*ko/ko*^ retinas at p15 and p17. The vertical bars mark the corresponding outer nuclear layer (ONL) thickness. (B) Quantification of photoreceptor layer thickness (REC+) of the different genotypes at p13, p15, p17, and p20. Each data point represents the mean photoreceptor layer thickness (n = 8 per genotype and time point) ± SEM; **p < 0.01, ***p < 0.001.

**Fig 2:**
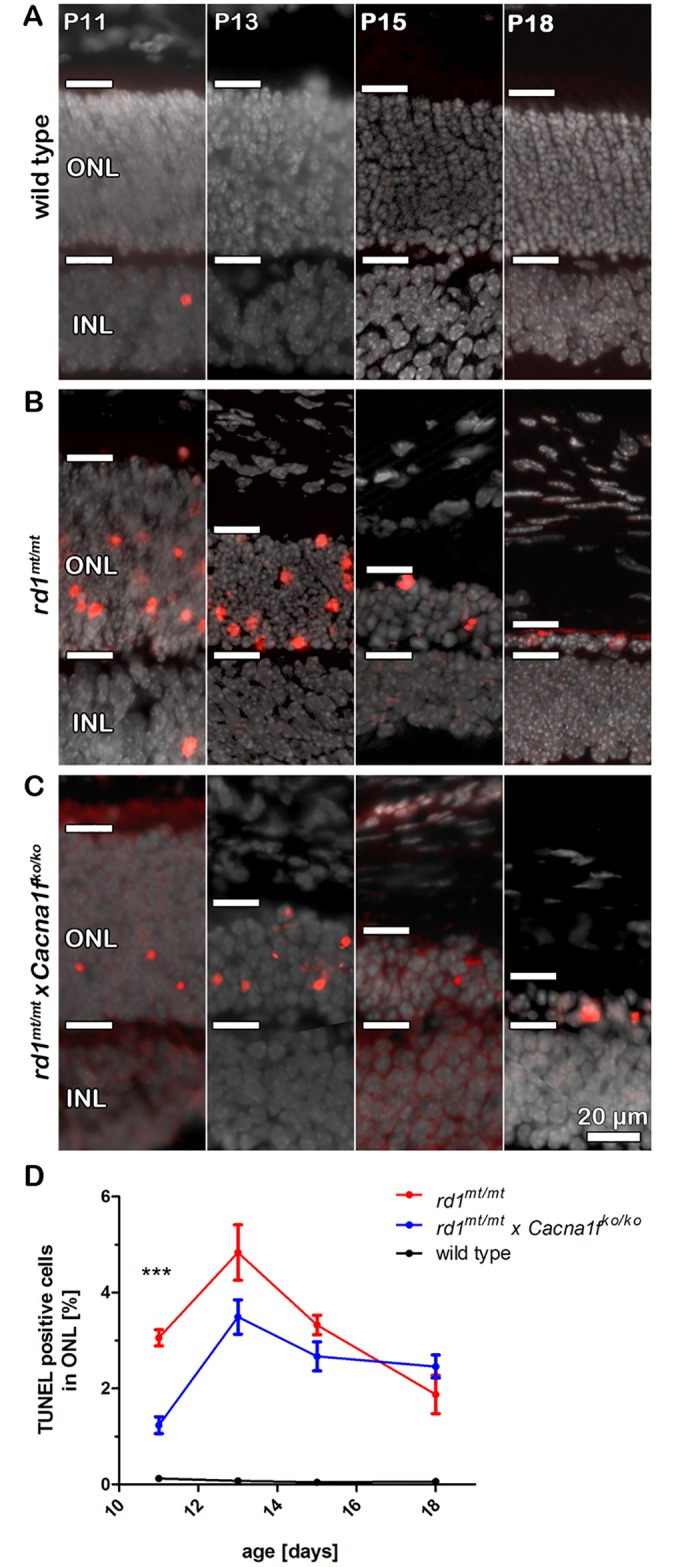
Knockout of *Cacna1f* reduces the number of dying photoreceptors in *rd1*^*mt/mt*^ mice. (A-C) Representative vertical cryo-sections of (A) wild type, (B) *rd1*^*mt/mt*^ and (C) *rd1*^*mt/mt*^ x *Cacna1f*^*ko/ko*^ retinas at p11, p13, p15, and p18 stained with TUNEL (red) and DAPI (grey). The horizontal bars mark the outer nuclear layer (ONL) borders. (D) Summary graph showing the quantification of TUNEL for the different genotypes at p11, p13, p15, and 18. Each data point represents the mean percentage of TUNEL positive cells in the ONL (n = 3–8 per genotype and time point) ± SEM; ***p < 0.0001.

To determine the influence of the synaptic VGCC on photoreceptor degeneration, we crossbred *rd1*^mt/mt^ with *Cacna1f*^ko/ko^ animals to obtain *rd1*^mt/mt^ x *Cacna1f*^ko/ko^ double mutant mice. Repeated OCT measurements of the retina were used to determine the photoreceptor layer thickness (receptor plus, REC+, see Methods) (Fig 1A). Right after eye opening (p13), *rd1*^mt/mt^ and *rd1*^mt/mt^ x *Cacna1f*^ko/ko^ mice already showed a severely reduced photoreceptor layer thickness in comparison to wild type animals (Fig 1A). When comparing the two models, we found no significant difference in the photoreceptor layer thickness between *rd1*^mt/mt^ and *rd1*^mt/mt^ x *Cacna1f*^ko/ko^ mice at this stage. However, at later time points, the photoreceptor layer in *rd1*^*mt/mt*^ x *Cacna1f*^*ko/ko*^ was significantly better preserved compared to *rd1*^*mt/mt*^ mice (p < 0.001 at p15 and p < 0.01 at p17, two-way ANOVA, n = 8), (Fig 1A and 1B). This finding was corroborated by comparing the photoreceptor rows in vertical retinal cryo-sections stained with the nuclear dye DAPI (Fig 2). Quantification at p15 revealed significantly less photoreceptor rows in *rd1*^mt/mt^ (3.0 ± 0.4) compared to *rd1*^mt/mt^ x *Cacna1f*^ko/ko^ (4.2 ± 0.2) retinas (p < 0.05, Welch's t-test, n = 4–7) confirming the *in vivo* OCT imaging findings.

### *Cacna1f* knockout decreases the number of dying *rd1*^*mt/mt*^ photoreceptors before eye opening

Next, we applied TUNEL assays on vertical retinal cryo-sections to determine the extent of cell death in wild type, *rd1*^*mt/mt*^, and *rd1*^*mt/mt*^ *x Cacna1*^*fko/ko*^ mice. In comparison to the OCT data (Fig 1), the TUNEL assay provided important additional insights to the cell death kinetics before eye opening. At p11, both *rd1*^*mt/mt*^ and *rd1*^*mt/mt*^ *x Cacna1*^*fko/ko*^ mice had a similar number of photoreceptor rows in comparison to wild type mice (Fig 2A–2C). At this time, both single and double mutant retinas displayed high numbers of TUNEL positive photoreceptor nuclei. However, when comparing the two models, *rd1*^*mt/mt*^ mice showed significantly more TUNEL positive photoreceptors than *rd1*^*mt/mt*^ x *Cacna1f*^*ko/ko*^ mice (p < 0.0001, ANOVA, n = 4–8), (Fig 2A–2D). At later time points (p13, p15, and p18) no significant difference in TUNEL positive photoreceptors could be observed. Taken together, both the *in vivo* OCT and the *ex vivo* TUNEL data suggested a significant, but not sustained delay of photoreceptor degeneration in the double mutant animals.

### Calpain activation in degenerating photoreceptors depends on *Cacna1f*

**Fig 3:**
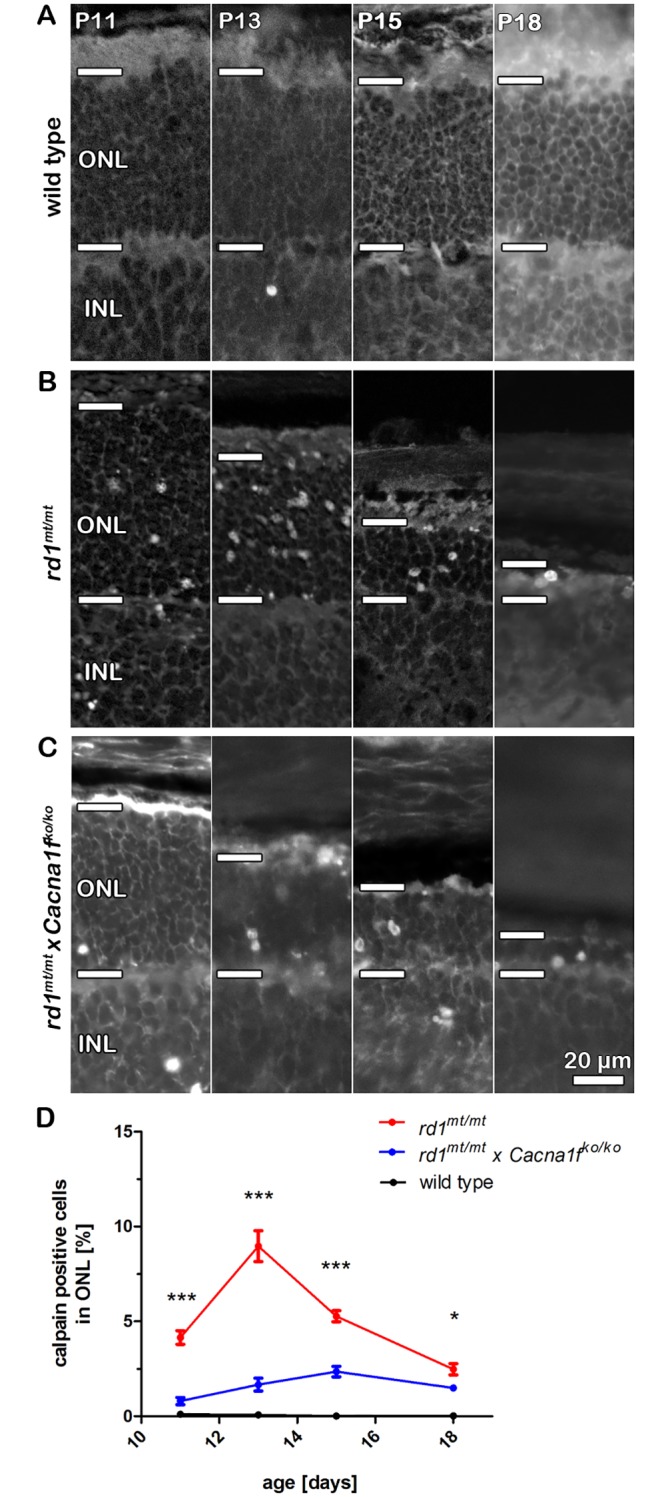
Knockout of *Cacna1f* significantly prevented excess activation of calpain in *rd1*^*mt/mt*^ photoreceptors. (A-C) Representative vertical retinal cryo-sections of (A) wild type, (B) *rd1*^*mt/mt*^ and (C) *rd1*^*mt/mt*^ x *Cacna1f*^*ko/ko*^ retinas at p11, p13, p15, and p18. Photoreceptor nuclei with increased calpain activity appear as grey dots or circles. The horizontal bars mark the outer nuclear layer (ONL) borders. (D) Summary graph showing the quantification of calpain-positive photoreceptors in the different genotypes at p11, p13, p15, and 18. Each data point represents the mean percentage of cells with increased calpain activity in the ONL (n = 3–8 per genotype and time point) ± SEM; ***p < 0.0001, *p < 0.05.

A hallmark of photoreceptor degeneration in *rd1*^mt/mt^ mice is the pronounced activation of the Ca^2+^ dependent protease calpain [26]. As described previously, calpain activation at least partially depends on Ca^2+^ entry through CNG channels in rod outer segments, since it is clearly reduced in *rd1*^*mt/mt*^ photoreceptors lacking the CNGB1 subunit of the rod CNG channel [14]. To test if Ca_v_1.4 channels also played a role in the activation of calpain in degenerating *rd1*^*mt/mt*^ photoreceptors, we analyzed calpain activity in our double mutant mice. When comparing *rd1*^*mt/mt*^ x *Cacna1f*^*ko/ko*^ mice with *rd1*^*mt/mt*^ mice, we found an almost complete lack of calpain activation in the absence of Ca_v_1.4 channels (Fig 3). At p11 the levels of calpain activity in double mutants were similar to wild type, while there was a significant difference to the enhanced calpain levels of *rd1*^*mt/mt*^ mice (p < 0.0001, ANOVA, n = 4–8), (Fig 3A–3D). At later time points (p13 –p18), calpain activity increased marginally in *rd1*^*mt/mt*^ x *Cacna1f*^*ko/ko*^ mice but never reached *rd1*^*mt/mt*^ levels (p < 0.0001 at p13 and p15, p < 0.05 at p18, ANOVA, n = 3–8).

## Discussion

Progressive rod and cone degeneration leads to functional blindness in RP patients. While the disease is still untreatable, various strategies emerge aiming at restoring the function or delaying the degeneration of photoreceptors. In order to provide a long-term beneficial effect a successful treatment has to achieve both, restoration of function and rescue of degeneration of rod photoreceptors.

One treatment strategy aims at decreasing intracellular Ca^2+^ levels to stop photoreceptor degeneration. Attempts to pharmacologically rescue photoreceptors in RP animal models by diltiazem have led to positive but contradicting findings [8–13]. Additionally, the Ca^2+^ channel blocker nilvadipine was tested in a clinical trial with RP patients resulting in promising but ambiguous effects on central visual field defects [27, 28].

In the present study we examined the role of the Ca_v_1.4 α1 subunit of the L-type VGCC by crossing *Cacna1f*-deficient mice with *rd1*^*mt/mt*^ mice. In addition to histological methods, we examined the mouse retina using *in vivo* OCT. This non-invasive imaging technique allowed for longitudinal examinations of the photoreceptor layer thickness in individual animals minimizing inter-individual variability. Our data on the deletion of the principal pore-forming Ca_v_1.4 α1 subunit of the L-type VGCC in *rd1*^*mt/mt*^ mice strengthen the view that synaptic Ca^2+^ entry through Ca_v_1.4 L-type VGCC contributes to rod photoreceptor degeneration. However, together with previous findings on the analysis of *rd1*^*mt/mt*^ x *Cancnb2*^*ko/ko*^ mice (15) the data show that genetic deletion of the photoreceptor L-type VGCC was not able to fully rescue rod photoreceptors from degeneration and resulted only in a short-term delay of degeneration.

Interestingly, our experiments uncovered a novel major role of L-type VGCCs on calpain activation during photoreceptor degeneration. Excessive activation of calpain-type proteases can be detected in a variety of different retinal degeneration models and was previously thought to be triggered by Ca^2+^ influx via CNG channels (3). Our data indicates that the synaptic VGCCs are at least equally important for calpain activation. In particular, VGCCs appear to be crucial for full activation of calpain-type proteases in *rd1* photoreceptors, providing evidence for a major role of synaptic Ca^2+^ entry in the excessive calpain activation commonly observed in degenerating photoreceptors.

The strong effect of *Cacna1f*-deletion on calpain activation in the *rd1*^*mt/mt*^ retina together with the temporally restricted protection of photoreceptors suggests that other L-type VGCC- and calpain-independent pathways dictate the long-term outcome in rapid photoreceptor degeneration. In addition, contribution of structural effects caused by the loss of L-type VGCC [21, 22, 29, 30] cannot be ruled out. Remarkably, when we compare our new data with the results of our previous study on the analysis of *rd1*^*mt/mt*^ mice lacking the CNGB1 subunit of the rod CNG channel [14], it is evident that CNG channel deletion has a relatively much stronger effect on *rd1* photoreceptor survival.

In light of these results and previous findings on the neuroprotective potential of Ca^2+^ channel inhibitors like diltiazem and dihydropyridines it seems preferable to simultaneously target multiple channels and signaling pathways to generate synergistic effects and hopefully provide enhanced and more sustained protection of photoreceptors in retinal degeneration.
